# Clinical implications of pharmacokinetics of sunitinib malate and N-desethyl-sunitinib plasma concentrations for treatment outcome in metastatic renal cell carcinoma patients

**DOI:** 10.18632/oncotarget.25423

**Published:** 2018-05-18

**Authors:** Kazuyuki Numakura, Nobuhiro Fujiyama, Makoto Takahashi, Ryoma Igarashi, Hiroshi Tsuruta, Atsushi Maeno, Mingguo Huang, Mitsuru Saito, Shintaro Narita, Takamitsu Inoue, Shigeru Satoh, Norihiko Tsuchiya, Takenori Niioka, Masatomo Miura, Tomonori Habuchi

**Affiliations:** ^1^ Department of Urology, Akita University Graduate School of Medicine, Akita, Japan; ^2^ Center for Kidney Disease and Transplantation, Akita University Hospital, Akita, Japan; ^3^ Department of Urology, Yamagata University, Faculty of Medicine, Yamagata, Japan; ^4^ Division of Pharmaceutical Science, Akita University Hospital, Akita, Japan

**Keywords:** renal cell carcinoma, sunitinib, N-desethyl-sunitinib, pharmacokinetics

## Abstract

In this study, we examined the association between the pharmacokinetics (PK) level of sunitinib malate (SU) and its metabolite N-desethyl-sunitinib (DSU) in terms of adverse events (AEs) and clinical outcomes in patients with metastatic renal cell carcinoma (mRCC). The PK of sunitinib (SU and DSU) was examined in 26 patients (20 men and 6 women) with mRCC. The associations between SU/DSU C0 and AE occurrence, best response rate, time to treatment failure, progression-free survival (PFS), and overall survival (OS) were investigated. Occurrence of grade 1 or higher hand-foot syndrome and thrombocytopenia (*p* = 0.002 and 0.024, respectively) was associated with a high concentration before morning intake (C0) level of SU. Low C0 levels of DSU were significantly associated with drug discontinuation due to disease progression (*p* = 0.035). Patients with DSU C0 level higher than 15.0 ng/mL showed a tendency toward increased PFS (61 weeks vs 12 weeks, *p* = 0.004) and OS (36 months vs 8 months, *p* = 0.040). The C0 level of SU and SU + DSU were not associated with prognosis. The higher level of C0 of SU may predict developing AEs and DSU C0 >15.0 ng/mL may lead to better prognosis of patients treated with sunitinib. PK of sunitinib may be useful for determining adequate dosages and prevention of severe AEs. Further studies are required to establish the utility of the PK of sunitinib in patients with mRCC.

## INTRODUCTION

Sunitinib has been widely used as an oral multi-targeted tyrosine kinase inhibitor for patients with metastatic renal cell carcinoma (mRCC) [1]. An initial dose-escalation study was conducted to determine the recommended dose, tolerability, basic pharmacokinetics (PK), and antitumor effects of 50 mg daily sunitinib given orally on a 4-week-on, 2-week-off schedule [1]. However, in a real clinical setting, this schedule would be intolerable for a substantial group of patients, especially Asians [2, 3].

After oral administration, sunitinib malate (SU) is primarily metabolized to its active metabolite (N-desethyl-sunitinib; DSU) [1]. Because DSU has an inhibitory profile similar to that of SU in plasma *in vitro* and has similar plasma binding proteins, the combination of SU plus DSU was estimated to represent the total active drug (total drug) [4]. No differences in PK have been observed between healthy volunteers and cancer patients in previous studies [5]. However, when analyzed across multiple studies, factors such as patient status, age, sex, race, body weight, and genetic background may affect the PK of SU in individuals, resulting in increased or decreased exposure to SU, DSU, or total drug. In fact, the necessity for SU dose individualization has gained increasing attention in clinical practice. Such a PK model may help improve the quantitative understanding of the complex PK of SU, DSU, and their determinants. Currently, PK of SU appears to be a useful tool for dose titration toward target C0 levels in patients that tolerate the C0-guided dose increase [6]. However, the impact of SU PK on clinical outcome remains unknown.

In this study, our objective was to clarify the usefulness of SU PK in mRCC patients. The association between PK of SU and DSU and clinical outcome was analyzed to explain the variability of clinical efficacy and adverse events following oral administration of SU.

## RESULTS

### General

Twenty-eight patients were enrolled in this study between August 2011 and July 2015. Twenty-six patients received at least 4 weeks of SU treatment and were assessed for treatment efficacy and toxicity (Table 1).

**Table 1:** Clinical characteristics of patients

Number of patients	26 (100%)	Number of metastatic sites	
Gender		1	10 (38%)
Male	20 (77%)	2	6 (23%)
Female	6 (23%)	≥3	10 (38%)
Age		Site of metastasis and recurrence	
Median	67	Lung	15 (58%)
Range	40–85	Lymphonode	13 (50%)
Histology		Bone	11 (42%)
Clear cell	24 (92%)	Liver	8 (31%)
Papillary	1 (4%)	Contralateral	7 (27%)
Translocation	1 (4%)	kidney	
Prior nephrectomy		Local	4 (15%)
Yes	21 (81%)	Others	3 (12%)
Diagnosis to therapy <1 year		Best response	
Yes	16 (62%)	CR	0 (0%)
Number of prior systemic therapy		PR	5 (19%)
0	14 (54%)	SD	18 (69%)
1	5 (19%)	PD	3 (12%)
≥2	7 (27%)	Reason for sunitinib discontinuation	
Detail of prior therapy		PD	16 (62%)
Cytokines	7 (27%)	AE	9 (34%)
Sorafenib	4 (16%)	Still continue	1 (4%)
Axitinib	6 (23%)	C0 level (ng/mL)(mean ± SD)	
Everolimus	2 (8%)	SU	76.7 ± 39.8
Karnofsky PS		DSU	16.8 ± 9.5
≥80	22 (85%)	SU+DSU	93.5 ± 45.4
<80	4 (15%)	AUC0-24 (mg/mL·h)(mean ± SD)	
MSKCC risk classification		SU	2.1 ± 1.1
Favorable	1 (4%)	DSU	0.5 ± 0.3
Intermediate	18 (69%)	SU + DSU	2.6 ± 1.2
Poor	7 (27%)		

Abbreviations: PS, performance status; MSKCC, Memorial Sloan-Kettering Cancer Center; CR, complete remission; PR, partial response; SD, stable disease, PD, progressive disease; AE, adverse event; C0, predose trough drug blood level; SU, sunitinib malate; DSU, N-desetyl sunitinib; AUC, area under the curve.

The median patient age was 67 (range: 40–85) years. All patients were Japanese and included 20 (77%) males and 6 (23%) females. Twenty-four (92%) patients had clear cell histology, one (4%) patient had papillary, and one (4%) patient had Xp11.2 translocation. Twenty-one (81%) patients underwent radical nephrectomy before starting systemic therapies. Fourteen (54%) patients were given SU as a first-line systemic therapy, 5 (19%) patients as a second-line therapy, and 7 (27%) as a third-line therapy. Twenty-two (85%) patients had Karnofsky PS 80 or greater and 4 (15%) patients had less than 80. Only 1 (4%) patient was classified as being at favorable risk by the MSKCC risk classification system, 18 (69%) patients as being at intermediate risk, and 7 patients as being at poor risk (27%). Ten (38%) patients had one metastatic site, 6 (23%) patients had two, and 10 (38%) patients had three or more.

### Pharmacokinetic analysis

**Figure 1:**
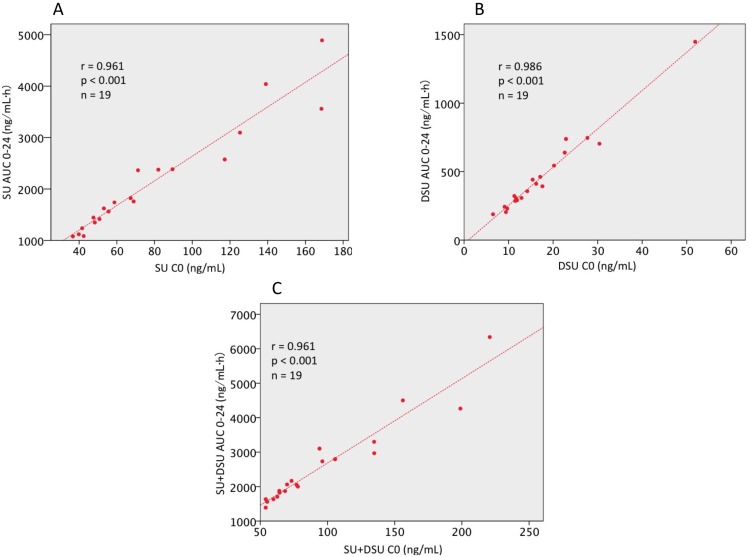
Correlation analysis between AUC_0-24_ and C0 in patients with mRCC The strong correlation between AUC_0-24_ C0 were confirmed in (**A**) sunitinib malate (r = 0.961, *p* < 0.001), (**B**) N-desethyl sunitinib (r = 0.986, *p* < 0.001), (**C**) total sunitinib (r = 0.961, *p* < 0.001). SU, sunitinib malate; DSU, N-desethyl sunitinib; SU + DSU, total sunitinib; AUC, area under concentration curve; C0, predose trough drug blood level; mRCC, metastatic renal cell carcinoma.

The mean dose of SU administered orally over the total duration of the study was 656.8 ± 117.8 μg/kg/day. Mean C0 and AUC_0-24_ values of SU and DSU were 76.7 ± 39.8 ng/mL, 2125.7 ± 1056.6 ng·h/mL and 16.8 ± 9.5 ng/mL, 463.3 ± 291.7 ng·h/mL, respectively. The correlation observed between C0 and AUC_0-24_ was very strong for SU, DSU, and total drug: r = 0.961, 0.986, and 0.961, respectively (Figure 1). Due to this, C0 was used for PK analysis because C0 blood collection needs to be done only once and at a clinically feasible time point.

### Antitumor effects

The mean follow-up duration after SU initiation was 20.6 (range: 2–50) months. At the time of analysis, only one patient continued taking SU. Sixteen patients ceased SU treatment because of disease progression, whereas 9 patients discontinued treatment because of AEs. Seventeen patients died at the time of analysis. Estimated median time to treatment failure (TTF), progression-free survival (PFS), and overall survival (OS) were 13 (range: 3–127) weeks, 33 (range: 4–127) weeks, and 15 (range: 1–50) months, respectively. The best response assessment was available for all patients (Table 1). None of the patients achieved a complete response, whereas 5 (19%) patients showed a partial response (PR), 18 (69%) showed disease stabilization, and 3 (12%) showed disease progression. Twelve (47%) patients needed to reduce SU dose because of AEs. Only one patient could tolerate a titrated SU dose up to 50 mg.

### Pharmacodynamic analysis

**Figure 2:**
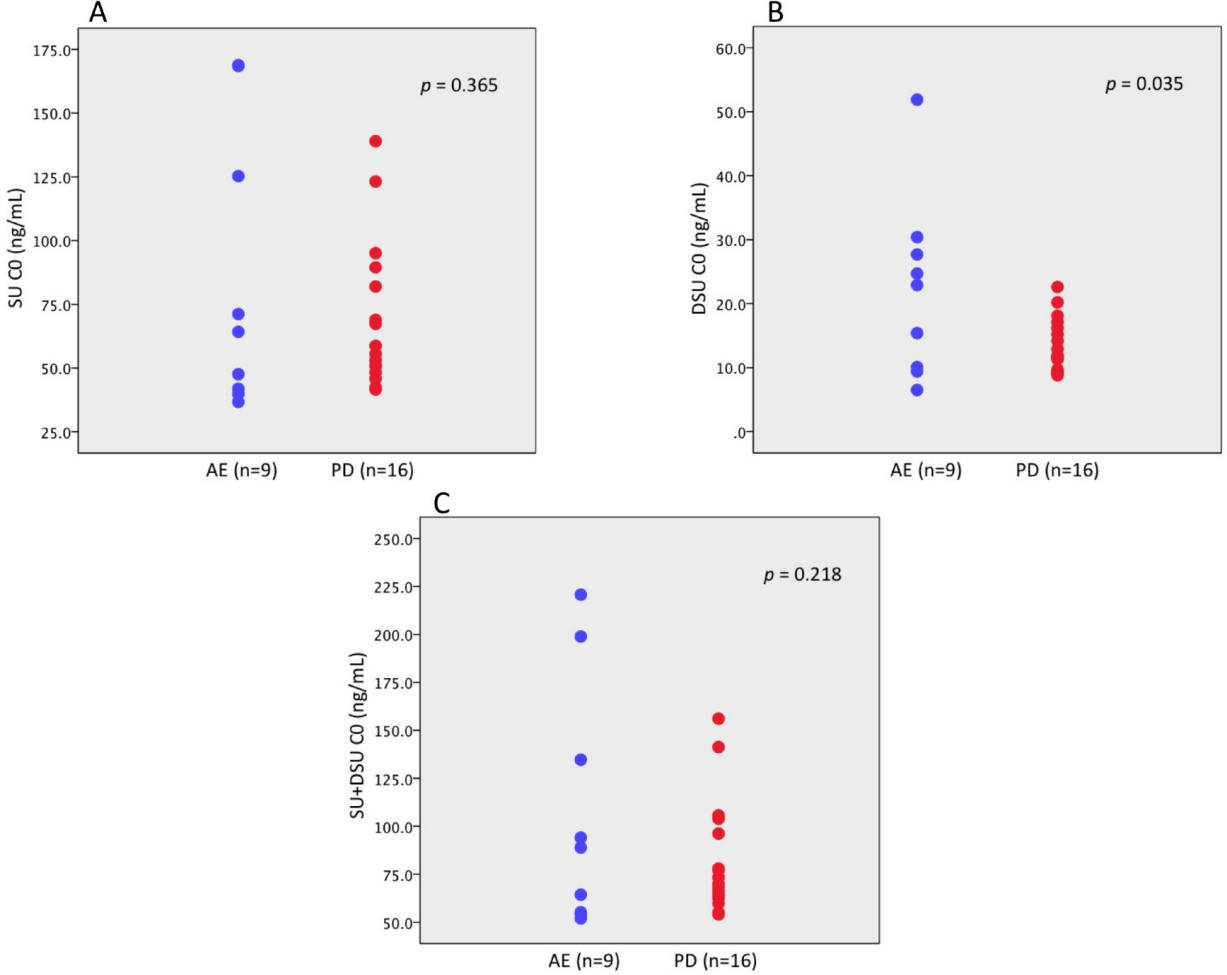
Comparison of C0 level of each drug in patients with mRCC (**A**) Sunitinib malate (AE 64.2 ng/mL vs PD 57.2 ng/mL, *p* = 0.365), (**B**) N-desethyl sunitinib (AE 22.9 ng/mL vs PD 12.4 ng/mL, *p* = 0.035), (**C**) total sunitinib (AE 88.9 ng/mL vs PD 71.7 ng/mL, *p* = 0.218). SU, sunitinib malate; DSU, N-desethyl sunitinib; SU + DSU, total sunitinib; C0, predose trough drug blood level; AE, adverse event; PD, progressive disease.

**Figure 3:**
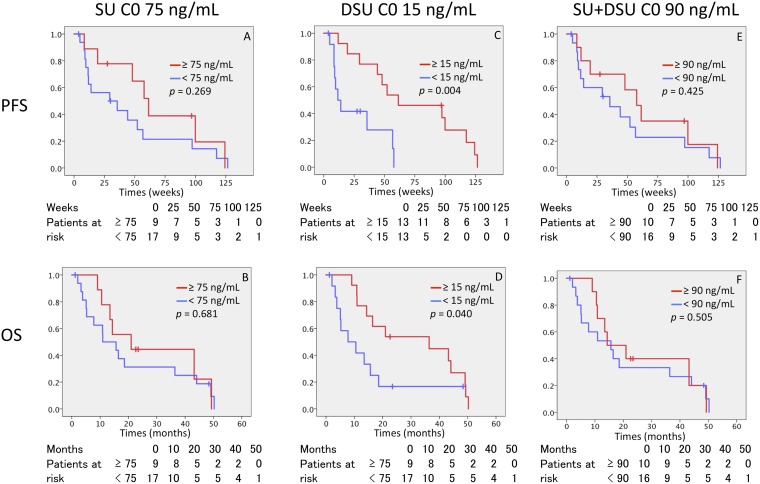
Kaplan–Meier curves for PFS and OS for C0 of SU, DSU, and SU + DSU (**A** and **B**) PFS and OS for SU C0 with cut-off value 75 ng/ml. (**C** and **D**) PFS and OS for DSU C0 with cut-off value 15 ng/ml. (**E** and **F**) PFS and OS for SU + DSU C0 with cut-off value 90 ng/ml. PFS, progression free survival; OS, over all survival, DSU, N-desethyl sunitinib; SU + DSU, total sunitinib; C0, predose trough drug blood level.

The lower C0 levels of DSU were significantly associated with drug discontinuation due to disease progression and were associated with worse tumor response (*p* = 0.035) (Figure 2). Moreover, DSU C0 did not reach 15 ng/mL in 13 patients. Of these patients, 10 patients (76.9%) discontinued sunitinib because of disease progression. The C0 levels of SU and total drug were not associated with prognosis. A receiver operating characteristic (ROC) curve was used to determine the optimal cut-off values of the SU C0, DSU C0, and total drug C0. The cut-off value had a maximum value of the Youden index (sensitivity+specificity − 1). The area under the curve was used to compare the performance between ROC curves. According to ROC analysis by patient survival, the cut-off value of drug plasma C0 level was 75 ng/mL (sensitivity 0.50, specificity 0.68), 15 ng/mL (sensitivity 0.50, specificity 0.45), and 90 ng/mL (sensitivity 0.50, specificity 0.59) on SU, DSU, and total drug, respectively. Patients with 15.0 ng/mL or higher C0 levels of DSU showed a longer PFS and OS compared to those with the C0 levels less than 15.0 ng/mL (61.4 weeks vs 11.6 weeks and 36.4 months vs 7.8 months, respectively), but no relationship between a C0 level of SU and total drug and treatment outcome was observed (Figure 3).

### Adverse events

The top five most common treatment-related AEs were fatigue in 17 (65%) patients, hypertension in 14 (54%), thrombocytopenia in 13 (50%), hand–foot syndrome in 13 (50%), and hypothyroidism in 9 (35%). Sixteen (62%) of 26 patients developed grade 3 or higher AEs, and the most frequent treatment-related grade 3/4 AEs among these patients was hypertension in 7 (27%), thrombocytopenia in 4 (15%), and hand-foot syndrome in 3 (12%). Although the occurrence rate of AEs was not affected by the C0 levels of DSU and total drug, the higher C0 levels of SU caused higher rates of AEs in HFS (sensitivity 61.5%, specificity 92.3%, positive predictive value 88.9%; *p* = 0.002) and thrombocytopenia (sensitivity 63.6%, specificity 86.7%, positive predictive value 77.8%; *p* = 0.024) when compared with the lower C0 levels of SU (Table 2).

**Table 2:** Relationships between C0 and adverse events

	Hand foot syndrome		Thrombocytopenia		Hypertention		Hypothiroidism		Fatigue	
	(G0 vs ≥ G1)		(G0 vs ≥ G1)		(G0 vs ≥ G1)		(G0 vs ≥ G1)		(G0 vs ≥ G1)	
	Median	*p*	Median	*p*	Median	*p*	Median	*p*	Median	*p*
SU C0	**48.3 vs 89.5**	**<0.01**	**50.8 vs 89.5**	**0.02**	54.8 vs 65.8	0.90	50.8 vs 71.2	0.08	49.6 vs 70.1	0.06
DSU C0	12.9 vs 15.2	0.88	11.9 vs 16.2	0.44	11.7 vs 15.3	0.82	11.7 vs 16.2	0.55	11.7 vs 15.7	0.64
SU + DSU C0	**64.2 vs 75.2**	**<0.01**	**64.4 vs 75.2**	**0.01**	67.2 vs 77.5	0.82	64.4 vs 94.1	0.07	64.3 vs 91.5	0.16

Abbreviations: G, grade; SU, sunitinib; C0, predose drug concentration; DSU, N-desetyl sunitinib.

## DISCUSSION

In this clinical study, the safety and feasibility of PK in mRCC patients treated with SU were evaluated. The SU starting dose of 37.5 mg (4 weeks-on and 2 weeks-off or 2 weeks-on and a week-off) was based on previous studies that investigated a modified dosing strategy for SU [7]. The steady-state PK parameters of SU were associated with clinical efficacy [8]. As both AUC and C0 level increase proportionately with dose, these parameters should correlate with each other. In our study, rigid relationships were observed between AUC_0-24_ and C0 in both SU and DSU. In general, a C0 value is a more useful PK marker than AUC or other PK parameters because assessing the C0 level involves taking one blood sample before the first dose. Hence, C0 would be better to use as the target plasma levels in clinical setting.

The higher C0 level of SU, not DSU, was associated with higher occurrence rate of HFS and thrombocytopenia in this study, while the higher C0 level of DSU was associated with better antitumor effects. These results might contribute to constructing a clinically beneficial PK strategy. Former investigators reported that a cumulative C0 level of SU and DSU was a predictor of SU pharmacodynamics [9, 6, 10]. However, no definitive study has assessed the anti-tumor effects of each substrate. Total quantity of DSU was smaller than SU, but the distinct absorption rate of DSU was estimated, which was found to be larger than SU, suggesting the possibility of a first pass effect of SU [11]. In healthy volunteers, the presence of DSU in blood was two or three times longer than that of SU. [5] DSU tends to have a greater accumulation in plasma compared to SU [12]. In this study, the DSU threshold is 15 ng/mL or more for achieving an optimal clinical effect on mRCC patients. It might be noted from the previous descriptions that possible drug prescribing strategies can be used. Based on our results and previous studies [13], when DSU-C0 was less than 15 ng/mL with tolerable toxicity, sunitinib dosage can be increased. When DSU-C0 was higher than 15 ng/mL with toxicity, sunitinib dosage can be reduced. There were six (23%) patients who can be modify sunitinib dosage. Compared with a toxicity-based dose-modifying approach, drug discontinuation or additional toxicities could be better avoided because the ideal C0 level of SU was attained before AEs occurred. No definitive study has been conducted using this approach, and our study suggests that this approach, or one based on toxicity, provides better dosing adjustments for patients [11, 14].

The use of a C0 level may provide a convenient method for monitoring systemic exposure to SU in a clinical setting, thereby allowing optimization of the dosing regimen to gain maximum efficacy and minimum toxicity [15]. Algorithms for therapeutic drug monitoring of tyrosine kinase inhibitors have not been proposed until now [1, 6, 16], and the result of this study can contribute to the current literature. Further insights on C0 level and interpatient PK variability during SU treatment are warranted to allow the creation of rational designs of the future PK of SU and DSU.

There are some potential limitations to this study. First, since this is a retrospective study, the treatment schedule, dose modifications, and radiological evaluation were not carried out based on strict protocols. Second, we only assessed PK parameters on day 7 after initiating SU. Other time frames of PK analysis, such as before starting the next course of SU or after stopping drug administration, may provide additional information that could contribute to the administration of a more ideal dosing schedule for individual patients. Finally, the main limitation of this study was the lack of statistical power, which might lead to type II errors due to the small sample size. Further studies on larger cohorts are necessary to validate the present findings.

Monitoring C0 can be a valuable strategy for maximizing treatment effectiveness and minimizing unnecessary drug toxicities. The C0 level of DSU 15 ng/mL or more could provide better anti-tumor effects in SU-treated mRCC patients.

## MATERIALS AND METHODS

### Eligibility criteria

Patients with histologically proven advanced RCC with all Eastern Cooperative Oncology Group performance status were included in this study. Patients were not eligible if they had failed to recover from the toxicity of previous chemotherapy, radiotherapy, immunotherapy, or surgery. Written informed consent was obtained from all patients, and approval from our institutional review boards was obtained.

### Pretreatment and follow-up examinations

Complete medical history, physical examination, Eastern Cooperative Oncology Group performance status, CBC with differential and platelet count, biochemical profile (including electrolytes, renal, and hepatic function, coagulation, pancreatic amylase, and lipase), urinalyses, and chest X ray were recorded before starting treatment and repeated during the therapy under the guidance of attending physicians. Toxicity was graded using the National Cancer Institute Common Toxicity Criteria version 2.0. Tumors were measured by computed tomography scans within 4 weeks prior to starting SU. After starting the drug, the assessment interval was scheduled for individual patients by attending physicians. Tumor response was evaluated using Response Evaluation Criteria in Solid Tumors guidelines.

### Drug administration

All patients were administrated 37.5 mg SU at the beginning of this study. SU was given continuously to almost all included patients according to a schedule of 4 weeks followed by 2 weeks off or 2 weeks followed by 1 week off. The choice of first-line and second-line systemic therapy was made on the basis of the PS, extent of disease, comorbidities, previous treatments, individual preferences, and availability of medication. Certain patients underwent metastasectomies. All AEs were graded according to the National Cancer Institute Common Toxicity Criteria version 4.0.

### Dose modified procedure

Dose-limiting toxicity (DLT) was defined as at least a grade 4 hematologic toxicity or grade 3 or more non-hematological adverse event that was considered related to the drug. Doses were escalated based on occurrence of DLT during the first course of treatment. If no DLT was seen in the first cycle, the dose was titrated up to 50 mg. Treatment delay was allowed for patients to recover from toxicities. Dose reduction to the 25 mg was authorized in response to DLT if the patient manifested clinical benefits. If intolerant toxicity occurred after dose reduction to 25 mg, treatment was ceased.

### Pharmacokinetic analyses

A day after initiating SU, serial blood samples were collected for determining SU and DSU concentrations prior to and 3, 6, 9, 12, 24 h after morning oral administration of SU. AUC and C0 of SU and DSU were included to investigate the PK relationships between systemic exposure and parameters.

Measurements of SU and DSU concentration were performed at MASIS Inc. Food & Drug Nano Analysis (Aomori, Japan). Briefly, separation of the analytes was achieved by using liquid chromatography-tandem mass spectrometry (LC-MS/MS) equipment which consisted of a Prominence HPLC system (Shimadzu, Kyoto, Japan) equipped with a Grand ODS-80Ts (150 × 2.0 mm, 5 μm, TOSHO, Tokyo, Japan) at 40° C and a TSQ Quantum Discovery MAX mass spectrometer system.

### Statistical analysis

TTF was calculated as the time between the initiation of SU treatment and definitive stop of SU due to intolerance, disease progression, or death. PFS was defined as the time between the initiation of SU treatment and disease progression or death, as confirmed by radiological images or obvious clinical manifestations of progressive disease (PD). OS was defined as the time between SU initiation and death. The patient record in the database was closed upon patient death or final follow-up. The Mann–Whitney *U* test was used to determine the differences in continuous values between groups. The chi-square test was used to examine the differences in categorical data. Spearman's rank correlation coefficient test was used to assess correlations between AUC 0-24 and C0, and all results were expressed as a correlation coefficient (r). TTF, PFS, and OS were stratified by the Kaplan–Meier method, and were tested with the log-rank test. Differences with a *p*-value less than 0.05 were considered statistically significant. The analysis was performed using SPSS version 24.0 statistical software (SPSS Japan Inc., Tokyo, Japan).
